# Self‐gated free‐running 5D whole‐heart MRI using blind source separation for automated cardiac motion extraction

**DOI:** 10.1002/mrm.30322

**Published:** 2024-10-09

**Authors:** Isabel Montón Quesada, Augustin C. Ogier, Masaki Ishida, Masafumi Takafuji, Haruno Ito, Hajime Sakuma, Ludovica Romanin, Christopher W. Roy, Milan Prša, Jonas Richiardi, Jérôme Yerly, Matthias Stuber, Ruud B. van Heeswijk

**Affiliations:** ^1^ Department of Diagnostic and Interventional Radiology Lausanne University Hospital (CHUV) and University of Lausanne (UNIL) Lausanne Switzerland; ^2^ Department of Radiology Mie University Hospital Tsu Japan; ^3^ Advanced Clinical Imaging Technology Siemens Healthineers International AG Lausanne Switzerland; ^4^ Woman‐Mother‐Child Department Lausanne University Hospital (CHUV) and University of Lausanne (UNIL) Lausanne Switzerland; ^5^ CIBM Center for BioMedical Imaging Lausanne Switzerland

**Keywords:** 5D whole‐heart cardiovascular MRI, cardiac motion extraction, free‐running, self‐gating, blind source separation

## Abstract

**Purpose:**

To compare two blind source separation (BSS) techniques to principal component analysis and the electrocardiogram for the identification of cardiac triggers in self‐gated free‐running 5D whole‐heart MRI. To ascertain the precision and robustness of the techniques, they were compared in three different noise and contrast regimes.

**Methods:**

The repeated superior–inferior (SI) projections of a 3D radial trajectory were used to extract the physiological signals in three cardiac MRI cohorts: (1) 9 healthy volunteers without contrast agent injection at 1.5T, (2) 30 ferumoxytol‐injected congenital heart disease patients at 1.5T, and (3) 12 gadobutrol‐injected patients with suspected coronary artery disease at 3T. Self‐gated cardiac triggers were extracted with the three algorithms (principal component analysis [PCA], second‐order blind identification [SOBI], and independent component analysis [ICA]) and the difference with the electrocardiogram triggers was calculated. PCA and SOBI triggers were retained for image reconstruction. The image sharpness was ascertained on whole‐heart 5D images obtained with PCA and SOBI and compared among the three cohorts.

**Results:**

SOBI resulted in smaller trigger differences in Cohorts 1 and 3 compared to PCA (*p* < 0.01) and in all cohorts compared to ICA (*p* < 0.04). In Cohorts 1 and 3, the sharpness increased significantly in the reconstructed images when using SOBI instead of PCA (*p* < 0.03), but not in Cohort 2 (*p* = 0.4).

**Conclusion:**

We have shown that SOBI results in more precisely extracted self‐gated triggers than PCA and ICA. The validation across three diverse cohorts demonstrates the robustness of the method against acquisition variability.

## 1 INTRODUCTION

Cardiac MR (CMR) imaging is a powerful and versatile diagnostic tool for the evaluation of cardiac anatomy, function, and viability.^1^ However, cardiac examinations are complicated by respiratory, cardiac and, to a lesser extent, bulk motion, which can result in artifacts in the images that can prevent them from being diagnostically useful. To avoid cardiac and respiratory motion artifacts, electrocardiogram (ECG) triggering and breath holding or respiratory navigators are traditionally used.^2^ Bulk motion can in turn be reduced through patient coaching, comfort, and restraints, as well as by shortening the scan duration. Still, the delays and time inefficiency induced by the need for triggering and gating lead to longer scan times, while their correct configuration requires specialized technologists.

Alternatively, self‐gating can be used to extract cardiac^3^ and respiratory^4^ motion and retrospectively separate or resolve the data into different cardiac and respiratory phases. To simplify and accelerate high‐resolution functional and anatomical cardiac MRI scans, a free‐running 3D radial framework with self‐gating was recently proposed.^5^ Here, cardiac‐ and respiratory‐motion‐resolved 5D whole‐heart images are reconstructed without the need for ECG triggering or respiratory navigators. In such fully self‐gated (SG) free‐running motion‐resolved 5D CMR, principal component analysis (PCA) has been used to extract the respiratory and cardiac motion states from repeated superior–inferior (SI) projections.^6^ From the SI projection matrix, a 1D time‐resolved signal for every spatial location along the SI and coil elements is obtained. Therefore, the challenge relies in determining which signal or combination represents cardiac and/or respiratory motion. PCA is used to reduce data dimensionality and redundancy assuming uncorrelated sources, thus facilitating the analysis and extraction of motion signals in the frequency ranges of respiratory and cardiac motion from SI projections. However, this is not always sufficient to identify and extract the correct cardiac triggers (i.e., the starting points of cardiac intervals) from all data, as cardiac and respiratory components can be merged together or corrupted by artifacts. This incomplete separation of cardiac and respiratory components might be explained by respiratory sinus arrhythmia (RSA), a physiologic phenomenon where the heart rate increases during inspiration and decreases during expiration.^7^ In some cases, PCA is likely unable to completely separate both physiological signals as the heart rate is driven by respiration, which can cause the extraction of components with mixed cardiac and respiratory frequency ranges. Thus, incorrectly identified triggers can cause the reconstruction of low‐quality images with insufficiently resolved motion.

Blind source separation (BSS) techniques might therefore be a useful addition to PCA, since they can be used to transform the input data (observed mixtures) into a set of components (source signals) that are not known a priori, thereby theoretically improving the robust extraction of the almost independent respiratory and cardiac motion states. In this study, we implemented two BSS methods: independent component analysis (ICA),^8^ which is routinely used for motion extraction,^9^, ^10^ and second‐order blind identification (SOBI),^11^ which evaluates the spectral content of the temporally correlated estimated sources. Several studies have compared the efficiency and reliability of these BSS techniques. SOBI was recommended as an alternative to ICA when the original unknown sources have different frequency spectra to exploit their temporal correlation,^12^ and was found more stable in estimating the source signals. In terms of computation efficiency, SOBI has previously shown to be more time‐efficient than ICA and used less allocated memory.^13^

The goal of this study was therefore to compare these BSS techniques for the automated extraction of cardiac self‐gating signals from three different free‐running CMR datasets obtained in healthy volunteer and patient cohorts to ascertain their performance in different noise and contrast regimes. The BSS techniques were also compared to PCA, which served as a reference method since it has already been used in multiple self‐gated 3D cardiac acquisitions for cardiac motion extraction.^5^, ^14^, ^15^ We compared the extracted signals to the gold‐standard ECG, quantified the impact of the extracted triggers on the sharpness of the final images and determined the precision and robustness of the techniques for cardiac motion extraction in self‐gated free‐running 5D whole‐heart MRI.

## 2 METHODS

### 2.1 Study cohort and acquisition protocol

Three different cohorts were retrospectively analyzed in this study to compare the robustness of the proposed motion extraction methods in different noise, contrast, and artifact regimes (Table 1). All experiments were approved by the local ethics committees (Ethics Committee of the Canton of Vaud (CER‐VD) for Cohorts 1 and 2; Clinical Research Ethics Review Committee of Mie University Hospital for Cohort 3). For Cohorts 1 and 2, written and informed consent for the retrospective use of the data was given by all participants. For Cohort 3, individual consent was waived by the local ethics committee.

**TABLE 1:** Acquisition characteristics for the three different cohorts.

Dataset	N	Contrast agent	Magnetic field strength (T)	FOV (mm)	Base resolution	Acquisition time (min)	TE/TR (ms)	Flip angle (°)	Sequence type
Cohort 1	9	Native	1.5 (Aera)	220	160	5	1.76/3.64	67	bSSFP
Cohort 2	30	Ferumoxytol	1.5 (Sola)	220	192	6	1.64/2.84	15	GRE
Cohort 3	12	Gadobutrol	3 (Vida)	220	192	8	2.19/3.93	15	GRE

*Note*: All scanners made by Siemens Healthineers (Erlangen, Germany).

Abbreviations: bSSFP, balanced‐SSFP; GRE, gradient‐recalled echo; *N*, number of subjects per cohort.

**FIGURE 1:**
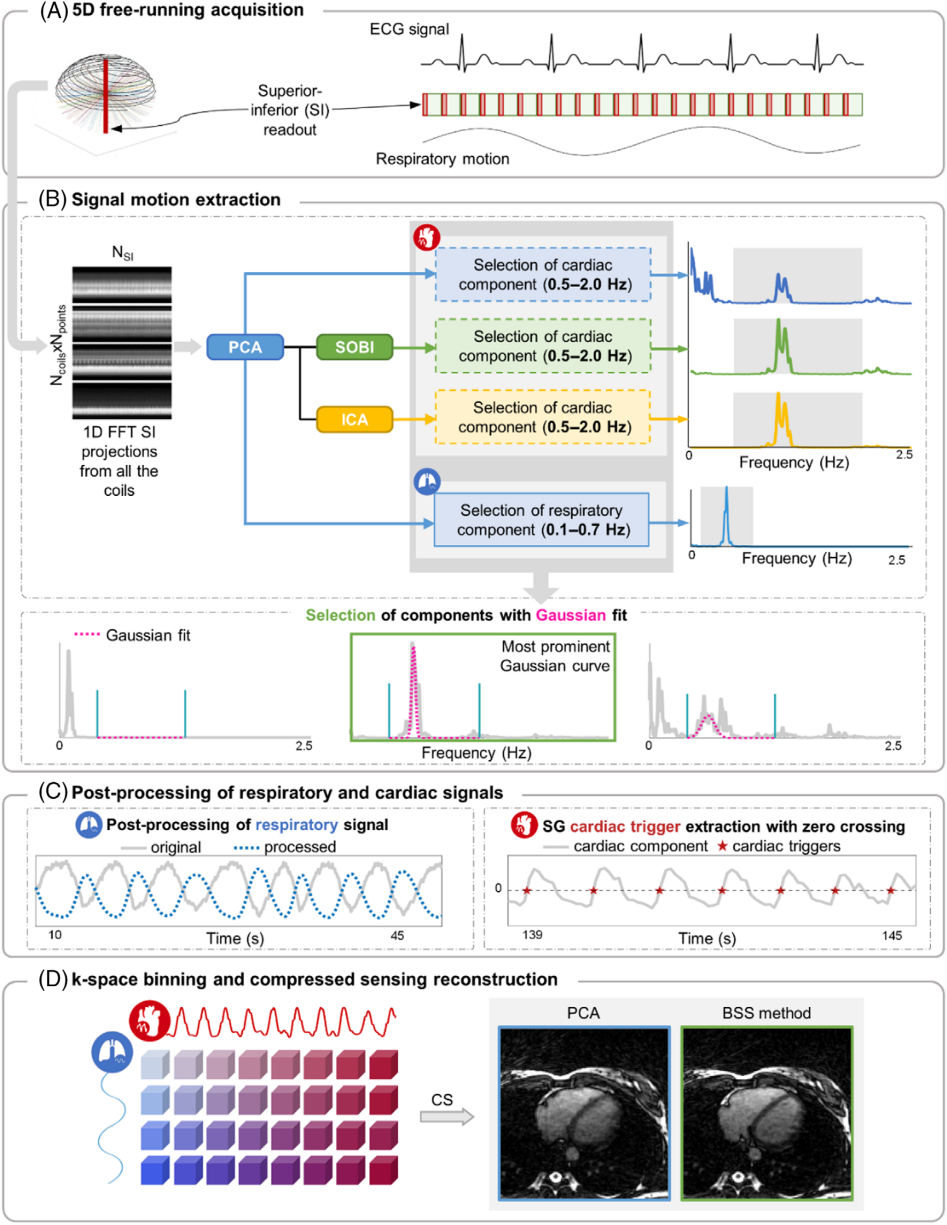
Overview of the motion extraction framework and image reconstruction. (A) Continuous acquisition of data using a 3D phyllotaxis trajectory with SI readouts (in red). (B) Cardiac signal extraction is performed by applying three methods to the SI projections: PCA, SOBI, and ICA. The selection of the components (here cardiac) is performed by automatically fitting a Gaussian (dotted pink line) to the component peaks and selecting the most prominent one (green square) in that frequency range (demarcated by blue lines). (C) The respiratory signal is detrended and filtered. The cardiac signal is used to extract the SG cardiac triggers (red stars) using a zero‐crossing approach. (D) Signal binning and CS reconstruction of the data in different respiratory and cardiac phases for PCA and the BSS method that yielded the lowest standard deviation with ECG.

In brief, these consisted of (1) 9 healthy volunteers (27 ± 2 y, 6 females) without known cardiovascular disease at 1.5T that were scanned with a balanced SSFP (bSSFP) sequence, (2) 30 congenital heart disease patients (12 ± 9 y, 14 females) that were scanned at 1.5T with a GRE sequence after injection with 2–4 mg/kg of ferumoxytol (Feraheme, AMAG Pharmaceuticals, Waltham, Massachusetts, USA) as a slow infusion over 15 min, as per clinical indication, and (3) 12 patients who were clinically referred for cardiac MRI for screening of coronary artery disease^14^ (70 ± 14 y, 2 females) and who were scanned at 3T with a fat‐suppressed^16^ GRE sequence after injection with gadobutrol (0.1 mmol/kg; Gadovist, Bayer Yakuhin, Osaka, Japan). All data were acquired with a free‐running pulse sequence that continuously sampled k‐space following a segmented 3D radial spiral phyllotaxis trajectory.^6^, ^17^ Each segment consisted of 22 lines, starting with a readout oriented in the superior–inferior (SI) direction for cardiac and respiratory self‐gating purposes (Figure 1A). For Cohorts 1 and 2, a 34‐element spine and chest receiver RF coil were used and for Cohort 3, an 18‐element body coil array and 72‐element spine coil array were utilized. Free‐running images were only acquired after contrast agent injection for Cohorts 2 and 3.

The ECG signal was recorded with a standard four‐lead vector ECG device (Siemens Healthineers, Erlangen, Germany) and used as the gold standard comparison for the extracted self‐gated cardiac signals.

### 2.2 Motion extraction

The SI readouts were used for signal extraction. After computing their 1D Fourier transform for all the individual RF coil elements, these were concatenated in a 2D matrix (Figure 1B). Due to the pseudo‐periodic sampling trajectory and eddy currents, trajectory‐related components modulated the self‐gated signals. To separate these components from the physiological motion components, angular dependence correction was applied before motion extraction, as previously described.^5^ PCA was always first applied to the 2D SI matrix to project the data in a lower dimensional space to simplify its processing by filtering out noise and whitening the data to make the components uncorrelated. This reduces computational costs and time and enhances the performance and convergence of subsequent BSS algorithms by providing a good initial separation of the data. Here, the matrix was reduced to a 10‐dimensional space as more than 90% of its variance could consistently be described by the first 10 principal components. The respiratory component was always first selected from this set of components. For cardiac motion extraction, three methods were compared (Figure 1B): (1) PCA as the reference method, (2) ICA,^18^ which is an independent component analysis algorithm that calculates the estimated independent components by maximizing the non‐Gaussianity of the input signals using an approximation of negentropy,^8^ and (3) SOBI,^19^ which is based on second order statistics and assumes temporally correlated sources with different spectral contents. The respiratory and cardiac components were selected based on their power spectral density within their coherent frequency ranges of physiological signals (respiratory 0.1–0.7 Hz, cardiac 0.5–2.0 Hz). The peaks in these frequency ranges were fitted with Gaussian curves, and the component with the most prominent curve was then automatically selected (Figure 1B). The respiratory signal was low‐pass filtered at 0.7 Hz to remove possible contamination from other sources. Afterward, it was linearly detrended and adjusted so that the inspiration phases corresponded to higher signal values, while the expiration phases corresponded to lower signal values (Figure 1C). The cardiac self‐gated triggers were detected from the cardiac signal component using a zero‐crossing approach, as previously described (Figure 1C).^5^

To assess whether respiration and heart rate were coupled, the average heartbeat during inspiration was compared to the averaged heartbeat during expiration.

### 2.3 Cardiac triggers comparison

First, missing ECG triggers were identified as subsequent cardiac trigger‐to‐trigger interval duration that exceeded 1.5 times the median duration of the 40 nearest neighbors. Conversely, intervals below 0.5 times the median were considered to be caused by false or extrasystolic triggers and also excluded for consistency. Due to the inherent delay in the SG cardiac triggers relative to the ECG R‐wave peaks, the SG and ECG trigger vectors were aligned by shifting the SG triggers by 10 ms increments to identify the alignment with the smallest average difference with the ECG triggers. Finally, SG and ECG‐adjusted cardiac trigger vectors were selected for further analysis and method comparison.

To evaluate the performance of the motion extraction methods, the cardiac intervals of the three sets of SG cardiac triggers (*S*) were compared to the cardiac intervals of the ECG triggers (*E*). The precision of extracted cardiac triggers was assessed by calculating the interval SD (ISD) of the absolute difference between the estimated SG cardiac intervals from consecutive triggers *S*_(*n+*1)_ − *S*_*n*_, and their corresponding ground‐truth ECG intervals *E*_(*n+*1)_ − *E*_*n*_:

(1) $$\mathrm{ISD}=\sqrt{\frac{1}{\left(N-1\right)}\sum_{n=1}^{N}\left[\left|\left(S_{n+1}-S_{n}\right)-\left(E_{n+1}-E_{n}\right)\right|-\mu^{2}\right]}.$$

The ISD is thus a quantitative measure of the inverse of the precision. The reference method PCA and the method that yielded the lowest ISD were retained for subsequent image reconstruction and sharpness analysis.

### 2.4 Motion‐resolved image reconstruction

The SG respiratory and cardiac components were used to order the continuously acquired data in respiratory and cardiac bins. Binning in the respiratory dimension was performed by dividing the respiratory signal into four equally populated phases (from end‐expiration to end‐inspiration) to separate the acquisition in the different motion states of the respiratory cycle. The cardiac triggers were used to sort the data in non‐overlapping phases as follows: firstly, the average cardiac interval was computed. Then, this average interval was divided by the desired bin width of 50 ms, resulting in the number of bins per cardiac cycle. Subsequently, each interval of the cardiac signal was divided into the previously calculated number of bins. Therefore, the number of cardiac bins depended on the subject's heart rate and ranged from 15 to 21 for Cohort 1, 14 to 20 for Cohort 2, and 11 to 26 for Cohort 3. No normalization or signal intensity correction was applied to the cardiac signals before cardiac binning, as only the cardiac triggers were used for binning purposes.

A k‐t sparse SENSE compressed sensing algorithm^20^, ^21^ was used to reconstruct 5D whole‐heart images by solving the following optimization problem (Eq. 2).

(2) $$\hat{x\;}=\underset{x}{\text{argmin}}\left({\left\|\mathrm{FCx}-y\right\|}_{2}^{2}+\lambda_{r}{\left\|\nabla_{r}x\right\|}_{1}+\lambda_{c}{\left\|\nabla_{c}x\right\|}_{1}\right)$$

where $\hat{x}$ is the reconstructed image, ***F*** and ***C*** are the non‐uniform Fourier transform operator and the coil sensitivity, respectively, per cardiac and respiratory bin, y is the k‐space raw data, and $\nabla_{r}$ and $\nabla_{c}$ are the first‐order differential operators along the respiratory and cardiac dimensions, respectively. The respiratory and cardiac regularization weights $\lambda_{r}$ and $\lambda_{c}$ were both set to 0.001. The optimization problem was solved using the Alternating Direction Method of Multipliers (ADMM) algorithm^22^ with 10 iterations. The image reconstruction time was recorded.

Images were reconstructed two times for each dataset: (1) using PCA as the cardiac motion extraction method and (2) using the most precise BSS method (Figure 1D).

A Linux workstation with two 24‐core CPUs (Intel Xeon Gold 6248R; Intel, Santa Clara, CA, USA), 1.5 TB of RAM, and dual Nvidia RTX A6000 GPUs (Nvidia, Santa Clara, CA, USA) was used to perform the offline reconstruction in MATLAB R2021b (The MathWorks, Natick, MA, USA). The reconstruction time ranged from 6 to 8 hours.

### 2.5 Image analysis

The sharpness of images resulting from each signal extraction algorithm was quantified by manually positioning points along the septal mid‐left‐ventricular blood pool‐myocardium interface in an axial orientation during end‐diastole and end‐expiration (Figure S1A). A cubic Bézier curve was fitted to pass through each of these points, and evenly distributed perpendicular lines were automatically placed along the curve. Next, a sigmoid function (Eq. 3):

(3) $$f\;\left(x;a_{0},a_{1},a_{2},s\right)=a_{2}+\frac{a_{1}-a_{2}}{1+10^{\left(a_{0}-x\right)s}}$$

where $a_{2}$ is the minimum intensity value, $a_{1}$ is the maximum intensity value, $a_{0}$ is the *x*‐value at which the function value is halfway between $a_{1}$ and $a_{2}$ and s the slope or steepness of the sigmoid, was fitted to these lines (Figure S1B),^23^ from which the 10%–90% rise distance (RD) in mm of the sigmoid was considered as a measure of sharpness for each image (Eq. 4).

(4) $$\mathrm{RD}=\frac{\mathrm{FOV}}{\mathrm{BR}}\left[f^{-1}\left(a_{2}+0.9\left(a_{1}-a_{2}\right)\;\right)-f^{-1}\left(a_{2}+0.1\left(a_{1}-a_{2}\right)\;\right)\right]$$

where FOV is the field of view, BR is the base resolution, and *f* ^*−1*^ is the inverse of *f* such that *f* ^−1^(*f*(*x*)) *= x*.

This distance indicates how quickly the intensity of the myocardium‐blood pool interface changes from low to high. A shorter rise distance indicates a steeper increase in intensity, which is associated with higher sharpness.

### 2.6 Statistical analysis

A one‐sided Bonett‐Seier test,^24^ a scale test for paired data, was used to test for lower dispersion between the three SG methods. Bland–Altman analysis and linear regression plots were also employed to evaluate the agreement between the SG and ECG cardiac intervals.

A paired Student's *t*‐test was performed to assess the difference in sharpness between the two selected methods and between average heart rate during inspiration and expiration. A *p*‐value lower than 0.05 was considered statistically significant.

## 3 RESULTS

### 3.1 Data rejection

For Cohort 1, all available datasets were used. For Cohort 2, the initial sample consisted of 58 datasets. However, a subset of 30 datasets was selected based on the number of missing ECG triggers. Patients with less than 0.75% of missing triggers (33 out of 58) were retained. Then, the ECG interval trace was inspected, and three more cases were discarded due to their low ECG quality (Figure S2). In Cohort 3, the initial sample consisted of 25 datasets. Due to the more common presence of magnetohydrodynamic effects and gradient‐induced artifacts at 3T, all ECG interval traces were examined and 12 out of 25 patients were selected based on their ECG quality.

### 3.2 Precision of cardiac trigger interval estimation

**FIGURE 2:**
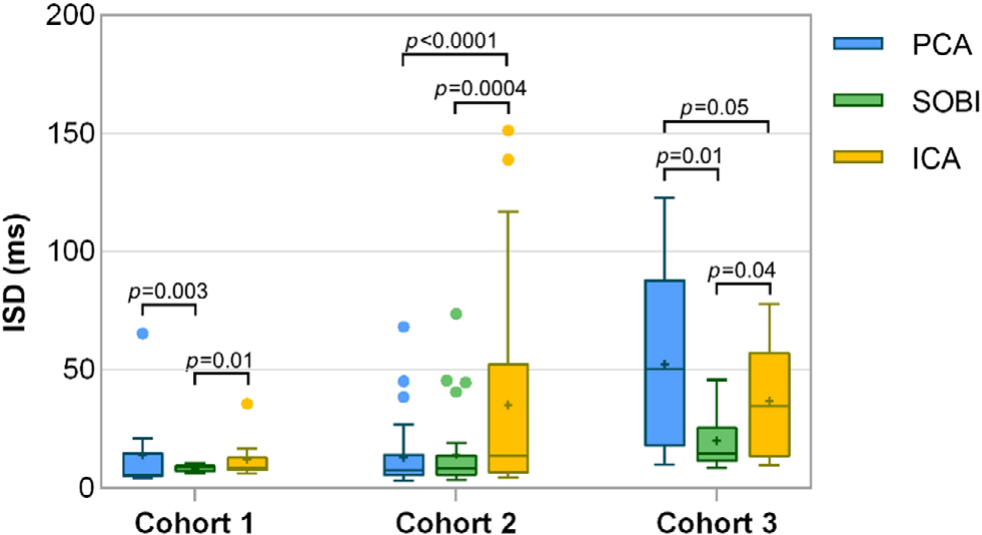
The precision of the motion extraction methods. ISD of self‐gated interval differences across three cohorts comparing PCA, SOBI, and ICA for cardiac motion extraction. In Cohort 1, employing SOBI after PCA resulted in a significant reduction in ISD values compared to PCA alone and notable advantage over ICA. No significant difference was observed between ICA and PCA (*p* = 0.2). For Cohort 2, while the ISD increased with SOBI after PCA, the differences were not statistically significant (*p* = 0.6). Both SOBI and PCA showed significant superiority over ICA. In Cohort 3, a significant decrease was observed with SOBI compared to PCA and ICA. A statistical difference was noted between ICA and PCA, with ICA demonstrating a slight superiority over PCA.

**FIGURE 3:**
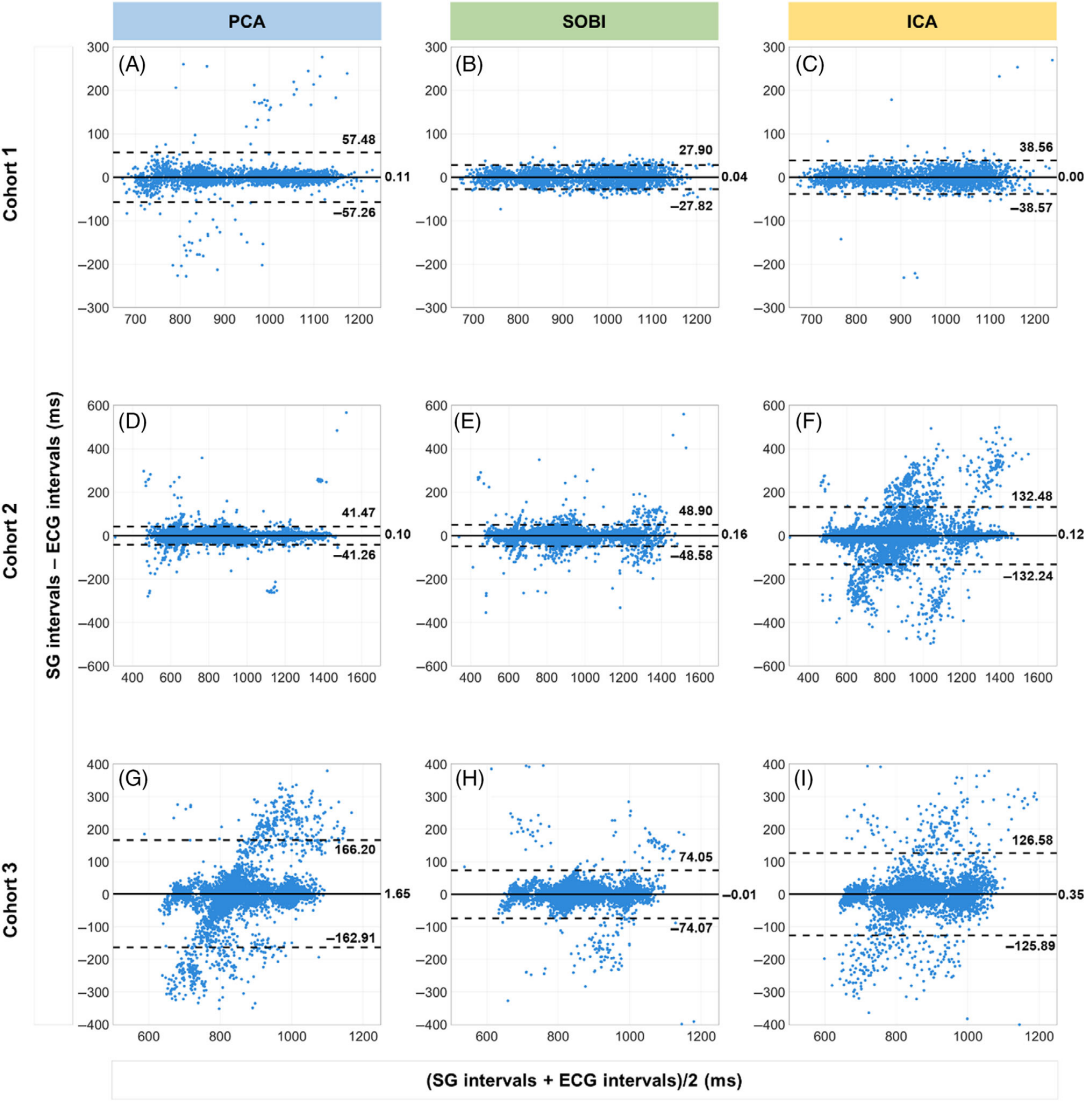
Bland–Altman analysis of the SG and ECG cardiac intervals. (A‐I) All individual cardiac intervals of a cohort are used in its respective plot. The black solid line indicates the mean difference, while the black dashed lines depict the 95% limits of agreement. The left column corresponds to the results using PCA as cardiac motion extraction algorithm. The middle and the right columns correspond to SOBI and ICA, respectively. SOBI generally showed the narrowest confidence intervals across Cohorts 1 and 3 (B, H, respectively) and the lowest number of outliers.

**FIGURE 4:**
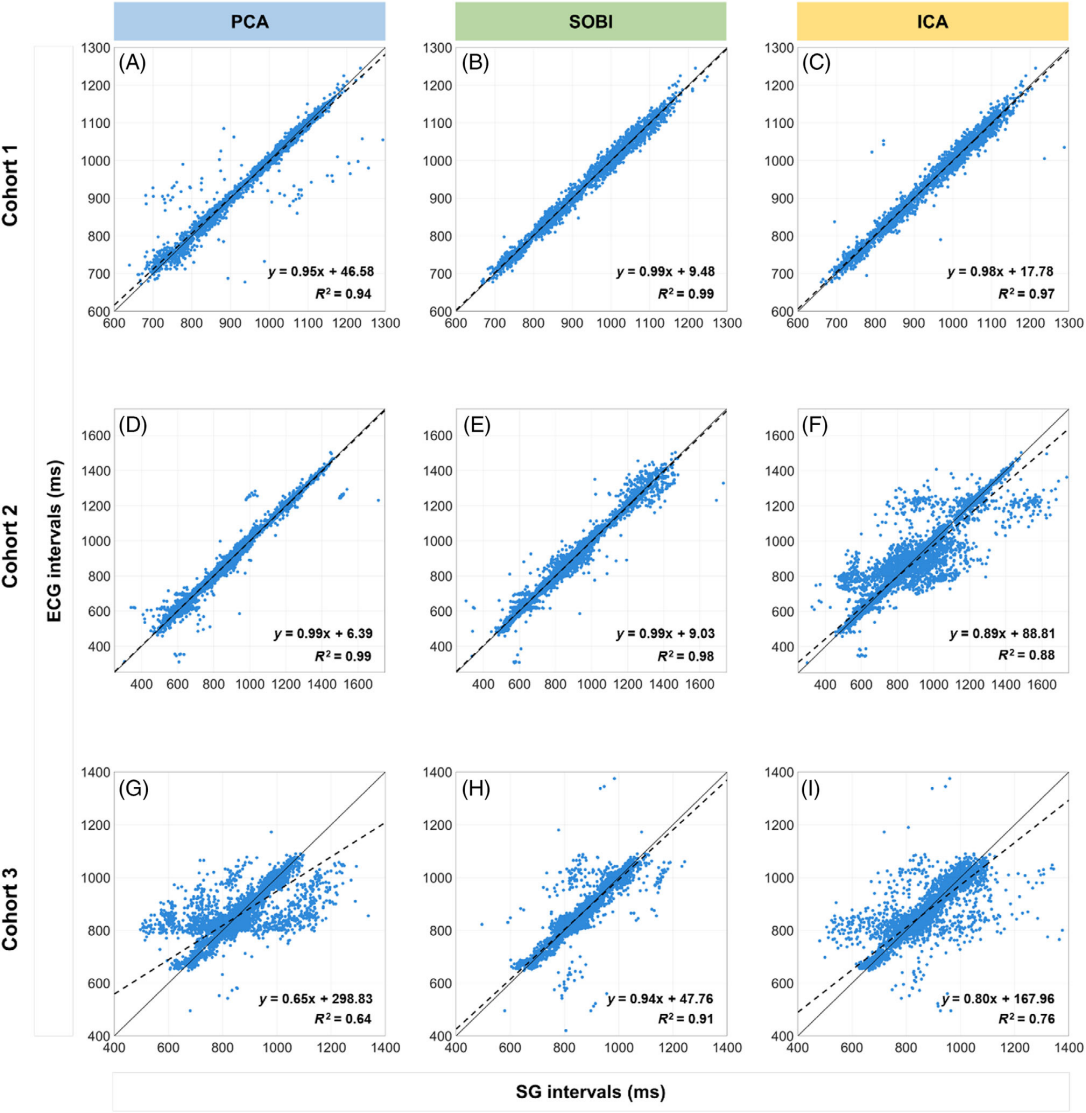
Linear regression between SG and ECG cardiac intervals. (A‐I) The dashed black line shows the linear fit of the data. The continuous black line refers to *y* = *x*. The linear fit and goodness of fit (*R* ^2^) for each graph is located on the lower right corner. A strong correlation can be observed in SOBI between ECG and SG cardiac intervals across all cohorts (B, E, H), with a high goodness of fit (*R* ^2^) in all cases.

In Cohort 1, the ISD of self‐gated cardiac interval differences was lower when employing SOBI compared to the other two techniques (ISD_PCA_ = 14.1 ± 18.8 ms, ISD_SOBI_ = 8.4 ± 1.5 ms, ISD_ICA_ = 12.3 ± 8.8 ms, all *p* < 0.01, Figure 2). Bland–Altman analyses (Figure 3A–C) demonstrated narrower confidence intervals for SOBI compared to ICA and PCA, while the linear regression plots similarly showed a higher linear correlation and goodness of fit *R* ^2^ for SOBI intervals, followed by ICA and PCA intervals (Figure 4A–C).

For Cohort 2, the ISD increased when applying SOBI after PCA instead of PCA alone (ISD_PCA_ = 13.2 ± 14.2 ms, ISD_SOBI_ = 14.2 ± 15.8 ms, Figure 2), although this was not statistically significant (*p* = 0.6). SOBI and PCA were more precise than ICA (35.2 ± 42.1 ms, *p* = 0.0004 and *p* < 0.0001, respectively). Bland–Altman analyses (Figure 3D‐F) illustrated similar confidence intervals for SOBI and PCA as well as a similar linear fit and goodness of fit *R* ^2^, while ICA again resulted in broader confidence intervals (Figure 4D‐F).

In Cohort 3, the ISD decreased with SOBI in comparison to PCA (ISD_PCA_ = 52.2 ± 37.1 ms, ISD_SOBI_ = 20.3 ± 12.1 ms, *p* = 0.01) and ICA (ISD_ICA_ = 36.9 ± 22.0 ms, *p* = 0.04, Figure 2). Bland–Altman analyses revealed narrower confidence intervals for SOBI compared to ICA or PCA alone (Figure 3G–I). Intervals extracted with SOBI also had a higher goodness of fit *R* ^2^ with ECG intervals in comparison to the two other methods as well as a significant linear relationship (Figure 4G–I).

In Cohort 1, the average heartbeat during inspiration was significantly different from the average heartbeat during expiration (66 bpm and 63 bpm, *p =* 0.01). No significant differences were found in Cohorts 2 and 3 (Table S3).

### 3.3 Sharpness of images

**FIGURE 5:**
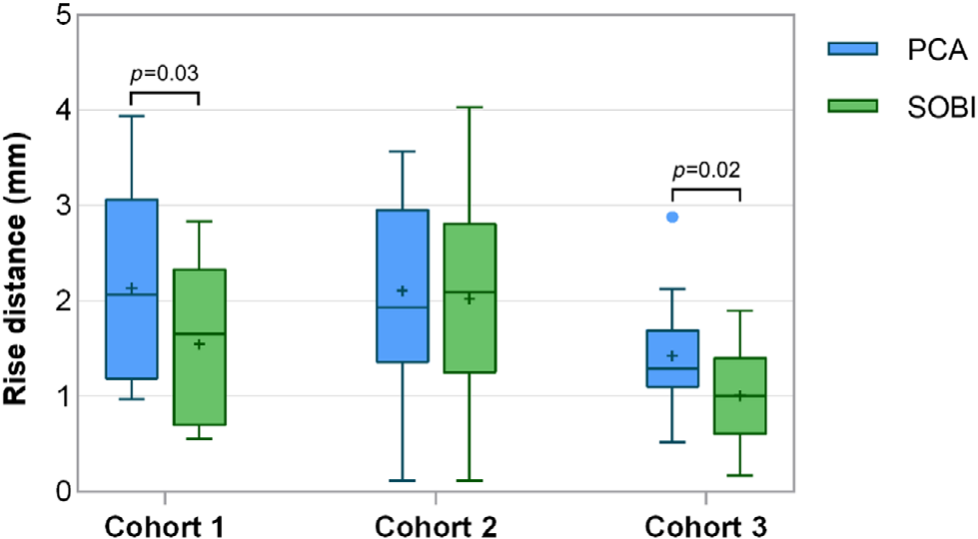
Image sharpness. In Cohort 1, SOBI demonstrated significantly lower rise distances compared to PCA alone. Similarly, in Cohort 3, a significant reduction in rise distance was observed with SOBI compared to PCA. However, Cohort 2 showed a non‐significant decrease in rise distance with SOBI compared to PCA (*p* = 0.4).

**FIGURE 6:**
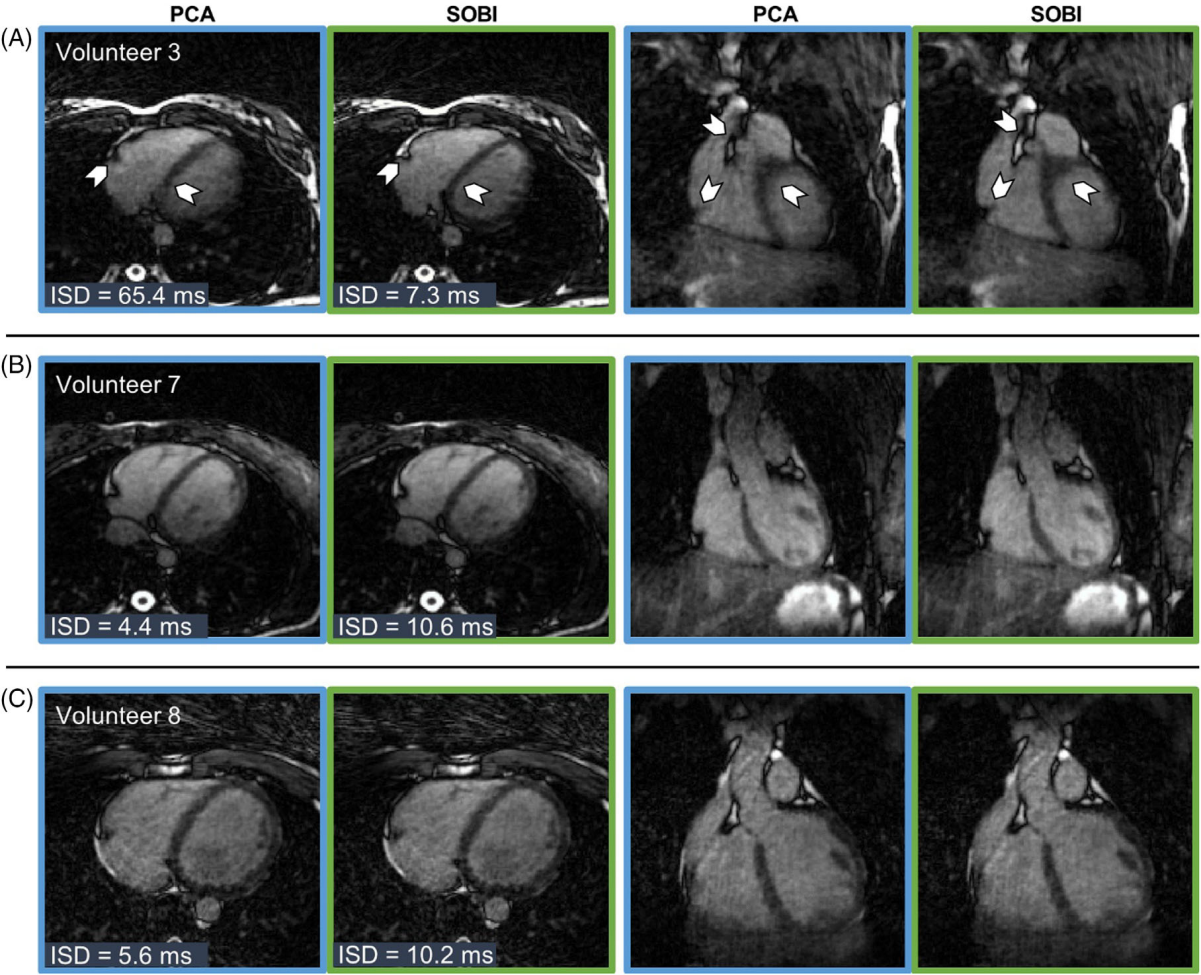
Images obtained with free‐running bSSFP in healthy volunteers from Cohort 1 without contrast agents. All transversal and coronal views are chosen to be in mid‐diastolic end‐expiratory phase. Images were reconstructed using PCA (blue outline) or using SOBI (green outline) as the cardiac motion extraction algorithm. (A) Volunteer 3 (27 y, female) where the PCA motion extraction resulted in the highest ISD (ISD_PCA_ = 65.4 ms, ISD_SOBI_ = 7.3 ms). The incorrect cardiac motion binning results in lower image quality (arrows) and sharpness. (B, C) The two cases in which SOBI resulted in the highest ISD. Despite the low precision, no difference can be observed in the reconstructed images compared to PCA (B, volunteer 7, 28 y, female and C, volunteer 8, 25 y, male).

**FIGURE 7:**
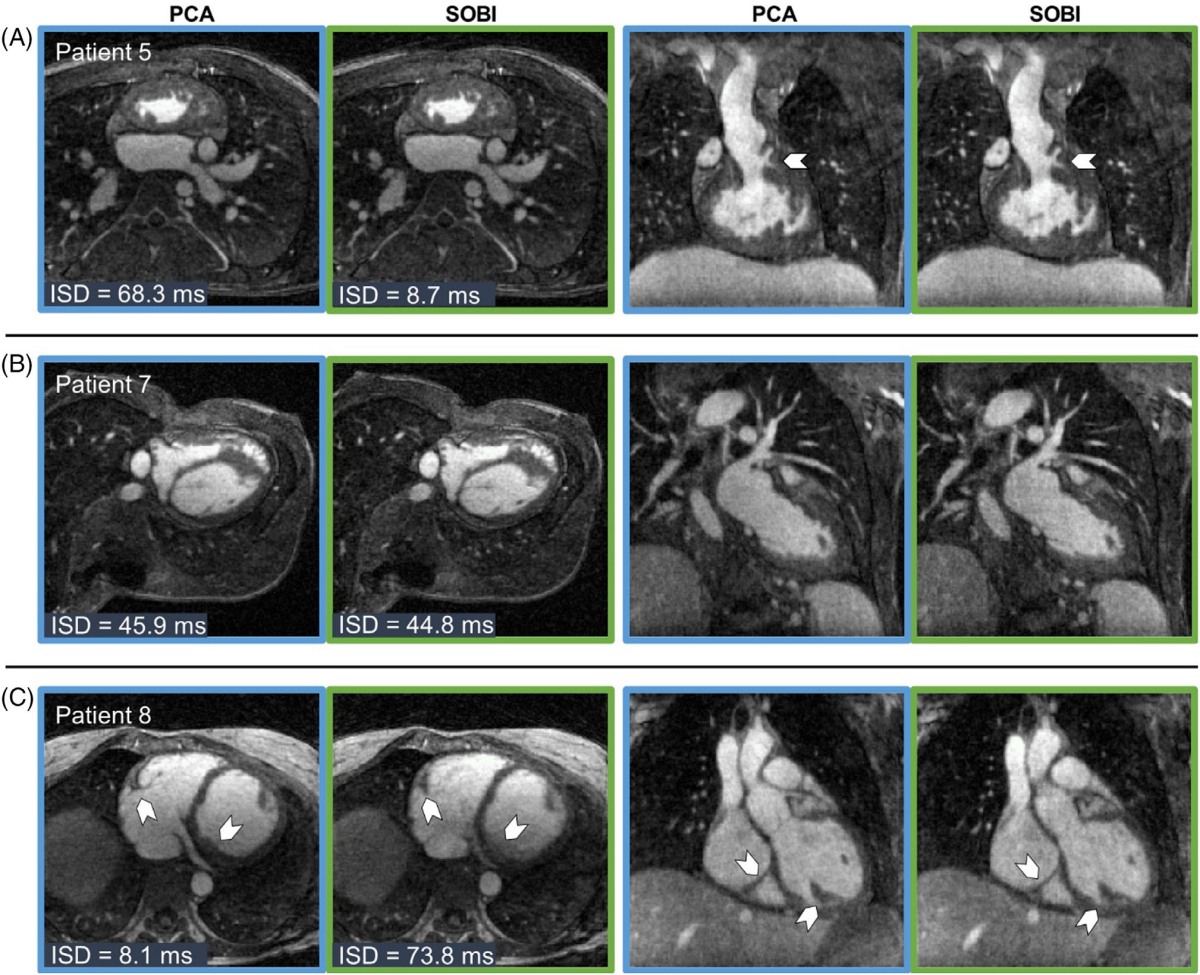
Free‐running 5D images from patients with congenital heart disease in Cohort 2. All transversal and coronal views are chosen to be in mid‐diastolic end‐expiratory phase. Images were reconstructed using PCA (blue outline) or using SOBI (green outline) as the cardiac motion extraction algorithm. (A) Patient 5 (right atrial isomerism, status post Fontan procedure, 23 y, male) in whom the visualization of fine details (arrows) improves in the case of SOBI (ISD_PCA_ = 68.3 ms, ISD_SOBI_ = 8.7 ms). (B) Patient 7 (status post Fontan procedure, 23 y, male) saw a similar ISD for both reconstruction methods, and no difference in image quality can be seen in the images. (C) In Patient 8 (Marfan syndrome, 44 y, female) SOBI resulted in the highest ISD of the cohort while PCA had a low ISD and, thus, resulted in sharper images (arrows).

**FIGURE 8:**
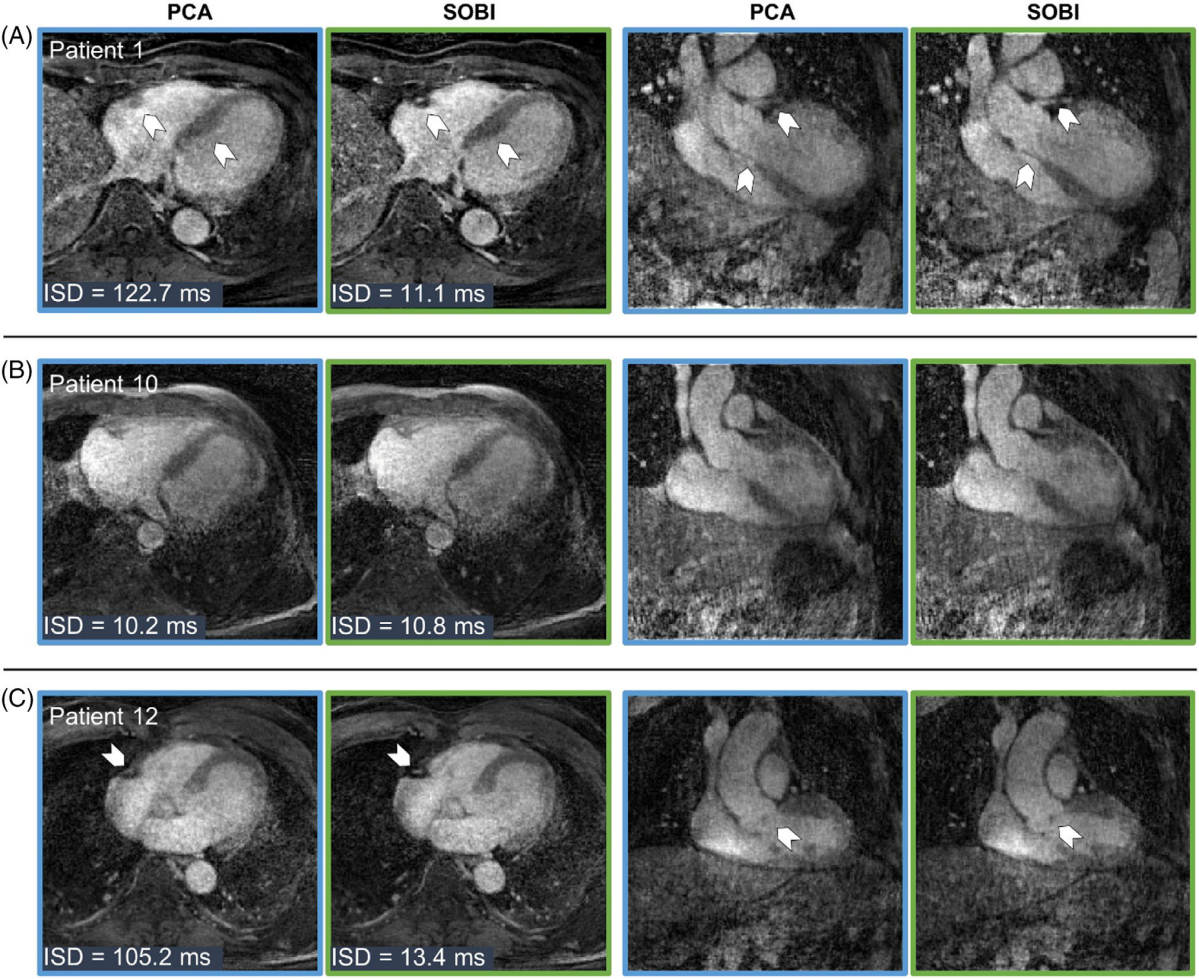
Free‐running 5D images from patients infused with gadobutrol in Cohort 3. All transversal and coronal views are chosen to be in mid‐diastolic end‐expiratory phase. Images were reconstructed using PCA (blue outline) or using SOBI (green outline) as the cardiac motion extraction algorithm. (A) Patient 1 (mild impairment of left ventricular function and dilated left ventricle, male, 70 y) in whom sharpness is improved using SOBI (ISD_PCA_ = 122.7 ms, ISD_SOBI_ = 11.1 ms). The right coronary artery can now be clearly visualized. (B) Patient 10 (myxopapillary ependymoma, male, 62 y) with comparable performance between PCA and SOBI and, thus, no discernible visual differences in the resulting images. (C) Patient 12 (vasospastic angina pectoris, male, 80 y) displays enhanced sharpness with SOBI compared to PCA due to the better extraction of the cardiac signal (ISD_PCA_ = 105.2 ms, ISD_SOBI_ = 13.4 ms).

The left‐ventricular blood‐myocardium sharpness was higher when using SOBI than when using PCA in all 3 cohorts (Figure 5). In Cohort 1, SOBI yielded a significantly lower rise distance (1.6 ± 0.8 mm) compared to PCA alone (2.1 ± 1.0 mm, *p =* 0.03). In Cohort 2, a non‐significant decrease in the rise distance was observed with SOBI (2.0 ± 1.0 mm) in comparison to PCA (2.1 ± 1.0 mm, *p =* 0.4). In Cohort 3, the rise distance was significantly reduced using SOBI (1.0 ± 0.5 mm vs. 1.4 ± 0.6 mm, respectively *p =* 0.02, Figure 6, Figure 7, and Figure 8, Video [Link], [Link], [Link]).

## 4 DISCUSSION

We developed a method for self‐gating cardiac motion extraction from free‐running 3D radial MRI using blind source separation techniques and successfully identified an algorithm (SOBI) that results in more precise trigger identification and higher image sharpness than the reference method. To demonstrate the robustness against acquisition variability of the self‐gating motion extraction framework, we analyzed a healthy volunteer and two patient cohorts with different acquisition protocols, age ranges, magnetic field strengths, contrast agents, and two different international centers. Therefore, related findings may help support the generalizability of the proposed algorithm.

In self‐gated 3D acquisitions, PCA has been the predominant method for extracting cardiac motion information from the SI projection matrix. Pang et al.^15^ developed a framework for 4D cardiac and respiratory self‐gated coronary MRA at 3T where the cardiac and respiratory motion components were extracted from the SI projection matrix of a 3D radial GRE sequence using PCA post contrast, while Di Sopra et al.,^5^ for a non‐contrast free‐running bSSFP acquisition at 1.5T, proposed to follow a similar approach. Bastiaansen et al. employed fast‐interrupted steady‐state (FISS) at 3T to enable a fat‐suppressed free‐running acquisition of the whole heart^25^ and PCA was used to extract cardiac motion components from the SI projection matrix. While they acknowledged the need for applying gradient spoilers to stabilize the SI projections for motion extraction at the expense of acquisition time, alternative methods for cardiac signal extraction were not explored. In contrast‐enhanced free‐running coronary angiography at 3T, PCA was again applied.^14^ Some studies among this broad range of PCA applications likely involved manual selection of PCA components to achieve optimal image quality, since in our multicentric retrospective study, we observed a high variability in the results when using automated PCA. Given previously published findings that demonstrated no statistically significant difference between the ECG‐gated and SG approaches in case of having a good quality ECG trace,^5^ reconstruction with ECG triggering was omitted in the present study.

ICA and SOBI have also already been used for cardiac self‐gating in MRI. Von Kleist et al. used ICA to extract cardiac motion for 2D cine images in a bSSFP acquisition with a Cartesian k‐space trajectory.^26^ They preferred ICA as the algorithm to compute the self‐gated cardiac triggers as it was less computationally expensive than SOBI. Nevertheless, SOBI has been successfully applied in studies involving cardiac and respiratory motion extraction using 2D Pilot Tone navigation under free‐breathing conditions^27^ and in cardiorespiratory motion‐tracking for 3D PET‐MR^28^ at 3T. In our case, we reduce the dimensionality of the data to a 10‐dimensional space before applying the BSS algorithms. In this way, the computational time was reduced significantly in comparison to analyzing all SI projections, making SOBI more suitable for the analysis. Moreover, ICA‐estimated components are not completely reproducible due to the random initialization of the gradient‐descent algorithm.^29^ As a result, the estimated sources can be different for every run, in contrast to PCA or SOBI, whose estimated components are always the same. Himberg and Aapo^29^ investigated a way to extract more reliable estimates by proposing ICASSO, an approach that iterates the ICA algorithm with different initial conditions to find a better estimation of the independent components by analyzing their clustering in the signal space, but this is more computationally expensive than ICA.

In our study, ICA did not improve upon PCA in the extraction of self‐gated cardiac triggers. One of the main disadvantages of ICA in the context of free‐running cardiac MRI data is that this algorithm is based on the independence of the estimated sources, and respiratory and cardiac motion are at least partially coupled due to RSA.^7^ SOBI however relies on the temporal correlation of the source signals and their different spectral contents, which is a representative feature of respiratory (0.1–0.7 Hz) and cardiac motion (0.5–2 Hz), and facilitates their separation. Several SOBI variants have been developed over time.^30^ However, considering the characteristics of the cardiac and respiratory source signals, the application of SOBI alone in this study proved to be sufficient.

Cardiac self‐gating is especially crucial at 3T, as the ECG can be deteriorated by gradient switching and the magneto‐hydrodynamic effect.^2^, ^31^ In fact, a higher ISD could be observed in all three methods in Cohort 3 compared to Cohorts 1 and 2. This outlier can likely be attributed in part to the quality of the ECG trace. Despite selecting a cohort with sufficient ECG quality for analysis, the difference between the SG and ECG intervals remained pronounced. We estimate that this observed increase in variability in ISD for the two patient cohorts cannot be attributed to the presence of contrast agents, but is rather caused by lower patient compliance and suboptimal ECG performance at 3T. Despite the thorough processing of the ECG traces, some errors are still expected in the patient cohorts compared to the traces obtained from healthy volunteers (Cohort 1). Cohort 2 consisted of pediatric CHD patients (12 ± 9 y), in which ECG traces are often corrupted by motion. This resulted in higher variability when comparing the SG and ECG intervals, leading to a higher ISD and ISD standard deviation. In Cohort 3, the increase was more pronounced due to patients being scanned in a 3T scanner. In this case, in addition to motion artifacts, the ECG signals could be deteriorated by gradient switching and the magneto‐hydrodynamic effect. Consequently, despite selecting a cohort with sufficient ECG quality for analysis, the discrepancy between the SG and ECG intervals remained significant. The importance of self‐gating acquisitions was highlighted in this study, as only half of the subjects of two cohorts could be analyzed due to the ECG trace quality. The remaining subjects lacked sufficiently consistent ECG signals to extract reliable cardiac triggers.

In the presence of contrast, in the case of Cohort 2, the similarity of SOBI and PCA ISDs were expected due to the very high blood‐myocardium contrast‐to‐noise ratio, which resulted in sufficient separation of the cardiac component from the noise and other sources of motion when applying only PCA. In the case of Cohort 3, SOBI still performed better than PCA only, since the contrast‐to‐noise ratio was not as high as in Cohort 2, which resulted in an incomplete separation of the components.

It is known that RSA diminishes in older populations and patients with cardiovascular pathologies, and it is stronger in athletes than in non‐athletes.^32^ In our case, we only found significant differences in the heart rate during expiration and inspiration in Cohort 1. Measuring RSA needs of high‐resolution cardiac and respiratory signals, along with specialized hardware and software for analysis, which was beyond the scope of this study. Rassler et al.^33^ studied the impact in RSA during functional MRI in a healthy volunteer cohort, and determined that in some cases, RSA was inverted and associated with the levels of anxiety and stress of the participants, which is influenced by the MRI environment itself and can alter normal breathing patterns and heart rate variability. Nevertheless, in several cases in our study, we could observe the failed separation of the respiratory and cardiac components when using PCA alone. This observation highlights the superior performance of SOBI in isolating the cardiac component from the respiratory signal, demonstrating its effectiveness over PCA in these instances.

This study provides insight into the varied performance of cardiac motion extraction algorithms in self‐gated free‐running 5D whole‐heart data in different contexts. However, it is important to acknowledge that while employing SOBI addresses certain challenges, it does not solve the inherent delay between the ECG and the estimated SG triggers. Consequently, visual detection of different cardiac phases in the reconstructed images was necessary. To address this limitation, efforts have been made to leverage artificial neural networks for the detection of the offset from the SG signal.^34^ Our method currently does not account for arrhythmias, which may be ignored or averaged out in the reconstruction process through the cardiac binning procedure explained in the Methods section. However, the analysis of varying cardiac interval lengths has been recently explored in 5D flow datasets,^35^, ^36^ examining the impact of arrhythmia on flow patterns. Similar to that approach, a future extension to our framework could involve incorporating a binning method that considers the histogram of cardiac interval durations.

## 5 CONCLUSIONS

In this study, we successfully developed a method for the precise automated extraction of self‐gated cardiac triggers from free‐running cardiac MRI using SOBI. In comparison to the reference method PCA and the other blind source separation technique ICA, it significantly improved both the precision of the cardiac trigger extraction and the sharpness of the resulting images. The method was validated in three different cohorts with diverse characteristics, demonstrating its robustness to different types of acquisition protocols, contrast agents, and field strengths.

## CONFLICT OF INTEREST STATEMENT

The PhD studies of L.R. are financially supported by Siemens Healthineers (Erlangen, Germany). M.S. receives non‐financial research support from Siemens Healthineers (Erlangen, Germany).

## Supporting information


**Figure S1.** An example of a sharpness measurement. (A) Manually selected points are depicted in red, while the interpolated Bézier curve is shown in yellow. Perpendicular lines that are automatically positioned along the curve and fitted with a sigmoid function for analysis, are highlighted in blue. (B) Example of a sigmoid fit. The points along the perpendicular line (blue) are fitted with a sigmoid function (green). The negative *x*‐axis values refer to the points at the left of the Bézier curve. The gray area indicates the distance from the 10% to the 90% points of the sigmoid curve.
**Figure S2.** Example of different recorded ECG cardiac interval durations. These traces were inspected for each case to decide whether the ECG could serve as a reference for this study. The range from 0.5 to 1.5 times the moving median limits are shown with green lines. Interval durations that fall above this band are considered to contain a missing ECG trigger and are indicated with red dots. The first row shows a high‐quality ECG trace, in which no missing or extra triggers can be observed. The second row shows a case in which missed trigger are detected, but that was included for analysis. The third row shows a case with highly variable intervals, indicating malfunctioning ECG triggering. This case was excluded from the analysis due to the low quality of the ECG cardiac interval trace.
**Table S3.** Heart rate during inspiration and expiration for the different cohorts.

**Video S4.** Cohort 1, healthy volunteer 3 without contrast agent. The case with the highest ISD for PCA (ISD = 65.4 ms, blue). The SOBI‐generated image (ISD = 7.3 ms, green outline) is sharper and contains fewer radial streaking artifacts.

**Video S5.** Cohort 2, ferumoxytol‐injected congenital heart disease patient 8. In this case, the SOBI extraction (green) yielded two components that contained frequencies in the cardiac range. The automatically selected component resulted in slightly lower image quality than the manually selected alternative. However, despite the presence of minor motion artifacts, the cardiac cycle remains discernible in the former reconstruction. When manually selecting the alternative component, SOBI performs as PCA (blue).

**Video S6.** Cohort 3, gadobutrol‐infused patient 1. The PCA‐guided reconstruction (blue outline) results in incompletely resolved cardiac motion.
