# Supplementary material for: Self‐gated free‐running 5D whole‐heart MRI using blind source separation for automated cardiac motion extraction
Source: Magn Reson Med. 2024 Oct 9;93(3):961–74. doi: 10.1002/mrm.30322 (PMC11680725; doi:10.1002/mrm.30322)
Supplement: Supplementary file 1 — Figure S1. An example of a sharpness measurement. (A) Manually selected points are depicted in red, while the interpolated Bézier curve is shown in yellow. Perpendicular lines that are automatically positioned along the curve and fitted with a sigmoid function for analysis, are highlighted in blue. (B) Example of a sigmoid fit. The points along the perpendicular line (blue) are fitted with a sigmoid function (green). The negative x‐axis values refer to the points at the left of the Bézier curve. The gray area indicates the distance from the 10% to the 90% points of the sigmoid curve. Figure S2. Example of different recorded ECG cardiac interval durations. These traces were inspected for each case to decide whether the ECG could serve as a reference for this study. The range from 0.5 to 1.5 times the moving median limits are shown with green lines. Interval durations that fall above this band are considered to contain a missing ECG trigger and are indicated with red dots. The first row shows a high‐quality ECG trace, in which no missing or extra triggers can be observed. The second row shows a case in which missed trigger are detected, but that was included for analysis. The third row shows a case with highly variable intervals, indicating malfunctioning ECG triggering. This case was excluded from the analysis due to the low quality of the ECG cardiac interval trace. Table S3. Heart rate during inspiration and expiration for the different cohorts. [file MRM-93-961-s004.docx]

**Supporting information**


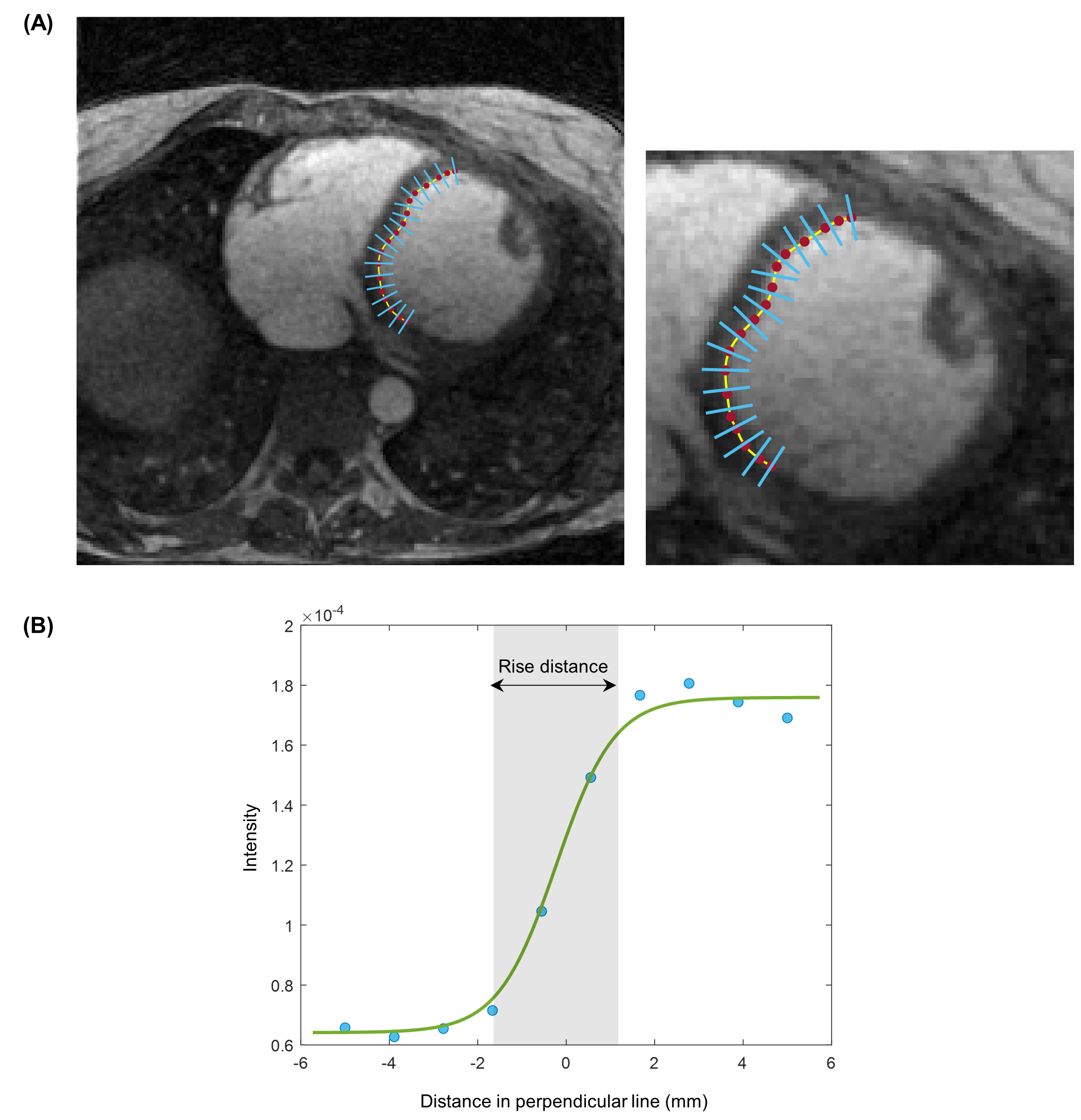


Supporting Figure S1. An example of a sharpness measurement. (A) Manually selected points are depicted in red, while the interpolated Bézier curve is shown in yellow. Perpendicular lines that are automatically positioned along the curve and fitted with a sigmoid function for analysis, are highlighted in blue. (B) Example of a sigmoid fit. The points along the perpendicular line (blue) are fitted with a sigmoid function (green). The negative x-axis values refer to the points at the left of the Bézier curve. The gray area indicates the distance from the 10% to the 90% points of the sigmoid curve.

**Tables**


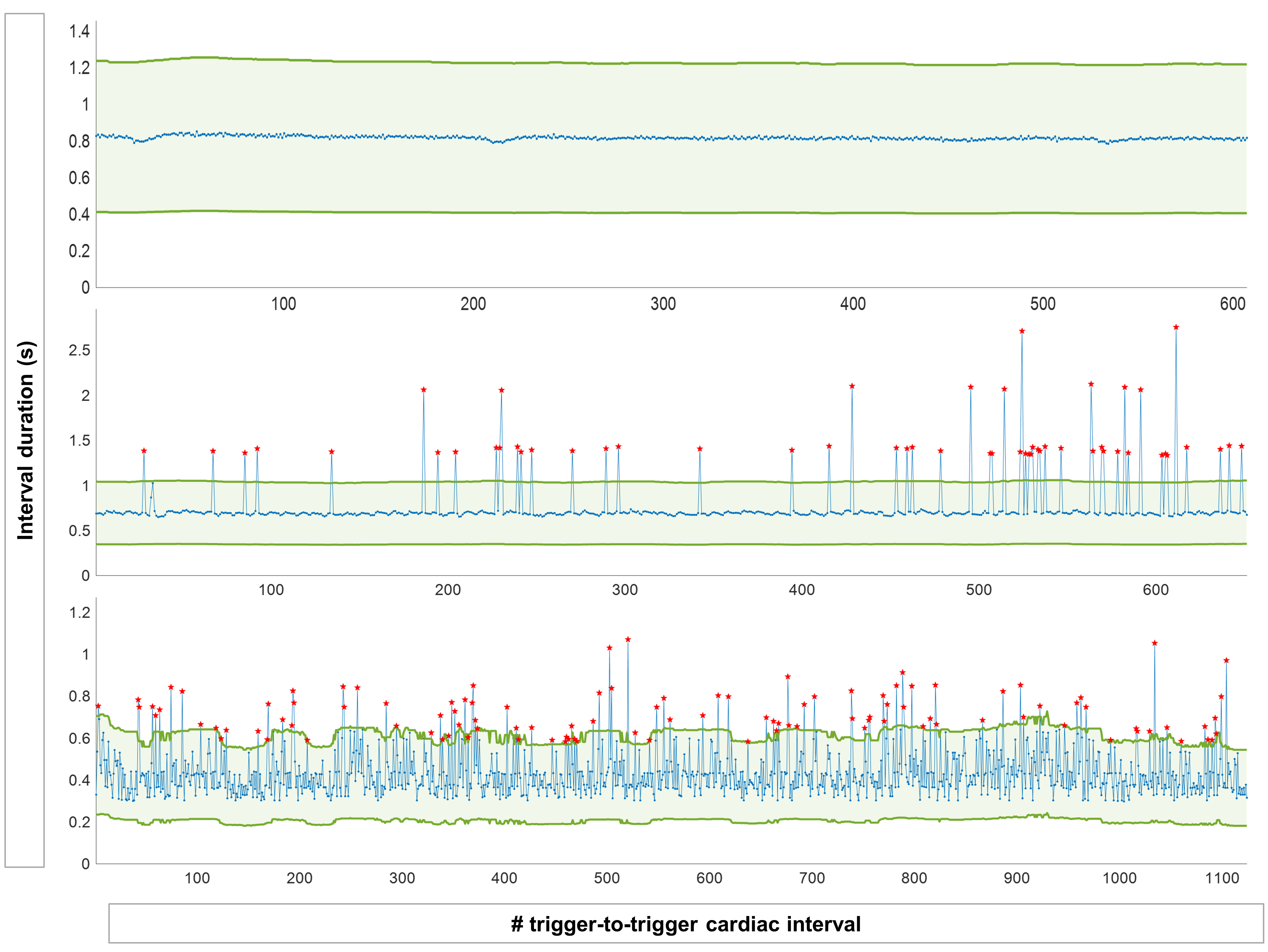


Supporting Figure S2. Example of different recorded ECG cardiac interval durations. These traces were inspected for each case to decide whether the ECG could serve as a reference for this study. The range from 0.5 to 1.5 times the moving median limits are shown with green lines. Interval durations that fall above this band are considered to contain a missing ECG trigger and are indicated with red dots. The first row shows a high-quality ECG trace, in which no missing or extra triggers can be observed. The second row shows a case in which missed trigger are detected, but that was included for analysis. The third row shows a case with highly variable intervals, indicating malfunctioning ECG triggering. This case was excluded from the analysis due to the low quality of the ECG cardiac interval trace.

Supporting Table S3. Heart rate during inspiration and expiration for the different cohorts.

| **Dataset** | **Inspiration (bpm)** | **Inspiration (bpm)** | **Difference** | ***p-*value** |
| --- | --- | --- | --- | --- |
| Cohort 1 | 66 ± 7 | 63 ± 8 | 3 ± 2 | 0.01 |
| Cohort 2 | 77 ± 15 | 78 ± 16 | -1 ± 2 | 0.2 |
| Cohort 3 | 71 ± 8 | 71 ± 8 | 0 ± 1 | 0.3 |
