# Supplementary figures and images for: Self‐gated free‐running 5D whole‐heart MRI using blind source separation for automated cardiac motion extraction
Source: Magn Reson Med. 2024 Oct 9;93(3):961–74. doi: 10.1002/mrm.30322 (PMC11680725; doi:10.1002/mrm.30322)

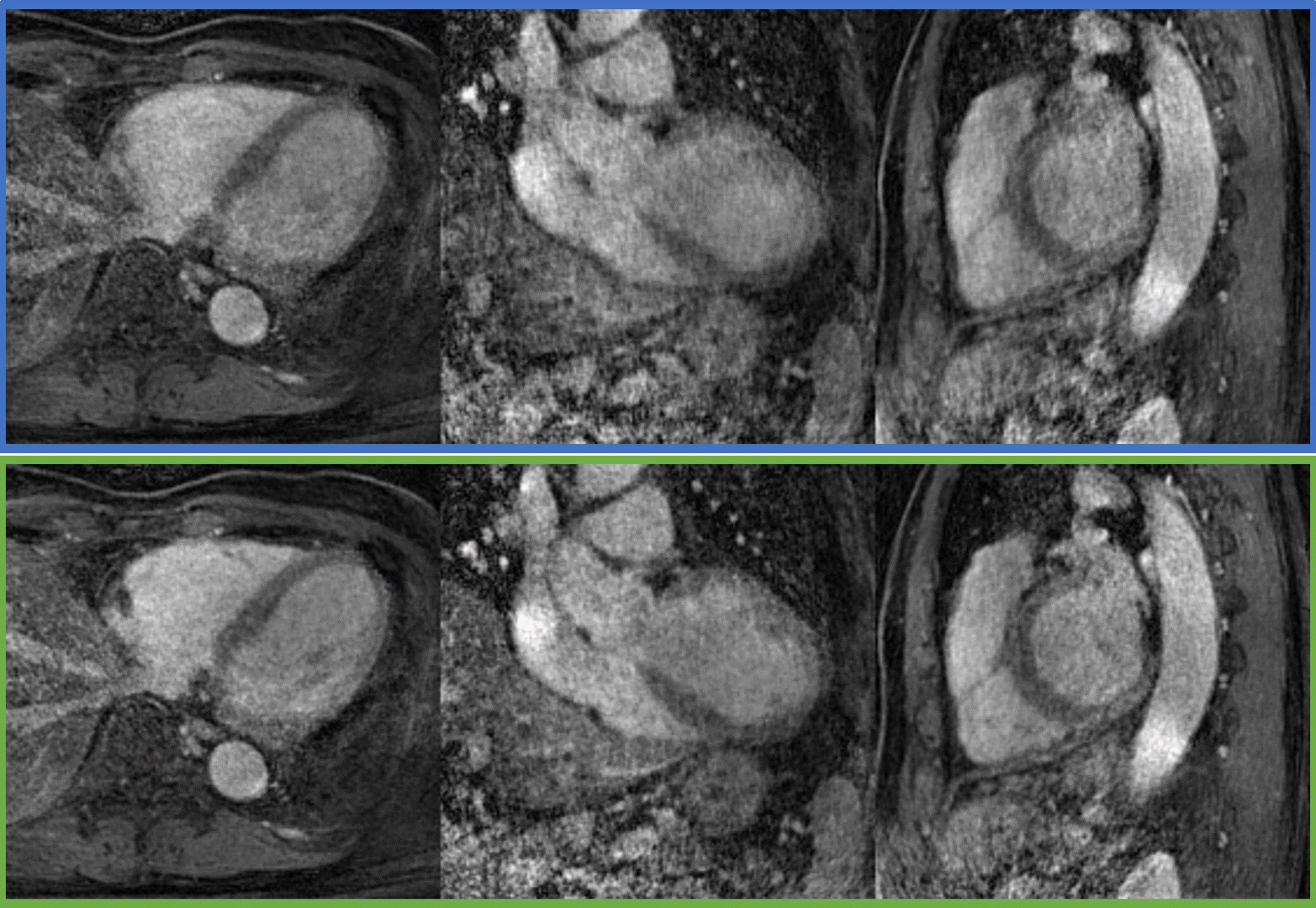

Supplement: Supplementary file 4 — Video S6. Cohort 3, gadobutrol‐infused patient 1. The PCA‐guided reconstruction (blue outline) results in incompletely resolved cardiac motion. [file MRM-93-961-s002.gif]
